# Vividness of Visual Imagery and Personality Impact Motor-Imagery Brain Computer Interfaces

**DOI:** 10.3389/fnhum.2021.634748

**Published:** 2021-04-06

**Authors:** Nikki Leeuwis, Alissa Paas, Maryam Alimardani

**Affiliations:** Department of Cognitive Science and Artificial Intelligence, Tilburg University, Tilburg, Netherlands

**Keywords:** brain-computer interface, motor imagery, BCI illiteracy, BCI performance, cognitive abilities, personality traits, vividness of visual imagery questionnaire, visuospatial memory and spatial ability

## Abstract

Brain-computer interfaces (BCIs) are communication bridges between a human brain and external world, enabling humans to interact with their environment without muscle intervention. Their functionality, therefore, depends on both the BCI system and the cognitive capacities of the user. Motor-imagery BCIs (MI-BCI) rely on the users’ mental imagination of body movements. However, not all users have the ability to sufficiently modulate their brain activity for control of a MI-BCI; a problem known as BCI illiteracy or inefficiency. The underlying mechanism of this phenomenon and the cause of such difference among users is yet not fully understood. In this study, we investigated the impact of several cognitive and psychological measures on MI-BCI performance. Fifty-five novice BCI-users participated in a left- versus right-hand motor imagery task. In addition to their BCI classification error rate and demographics, psychological measures including personality factors, affinity for technology, and motivation during the experiment, as well as cognitive measures including visuospatial memory and spatial ability and Vividness of Visual Imagery were collected. Factors that were found to have a significant impact on MI-BCI performance were Vividness of Visual Imagery, and the personality factors of orderliness and autonomy. These findings shed light on individual traits that lead to difficulty in BCI operation and hence can help with early prediction of inefficiency among users to optimize training for them.

## Introduction

Brain-computer interfaces (BCI) take brain activity as input and output an action that is carried out by an external device. This makes it possible for humans to interact with the environment without using their muscular system (Wolpaw et al., 2002). The electrophysiological signals of the brain are most commonly measured with electroencephalography (EEG). EEG is low-cost, non-invasive and user-friendly compared to other imaging techniques (Lee et al., 2019). BCI systems that rely on the execution of motor imagery (MI-BCI) demand users to imagine moving of a body part in order to cause a change in the brain activity of the motor cortex (Pfurtscheller and Neuper, 2001). The BCI system then learns to classify these changes and carry out a command accordingly (Thompson, 2019). This system can be assistive for motor-impaired patients (Mane et al., 2020) and elderly, but also has applications for healthy users (Belkacem et al., 2020).

Typically in MI-BCIs, the brain activity associated with motor imagery is recognized as event-related desynchronization (ERD) in the sensorimotor rhythms (SMR) that are characterized by the mu and beta frequency band over the sensorimotor cortex. This desynchronization is followed by an event-related synchronization (ERS) when motor imagery is over (Pfurtscheller and Da Silva, 1999). However, not everyone is capable of voluntary modulation of their brain activity in the way that is recognizable by the system (Allison and Neuper, 2010). Studies have indicated that fifteen to thirty percent of users are incapable of generating the proper brain activity even after training and thereby are unable to use a MI-BCI system. This lack of control is called “BCI illiteracy” (Allison and Neuper, 2010) or “BCI inefficiency” (Thompson, 2019).

The BCI inefficiency phenomenon is one of the biggest challenges in MI-BCI research, attracting interest in multiple previous studies. For instance, Lee et al. (2019) found that of all first-time users, 55.6% did not meet the proficiency threshold of 70% during the first session. Similarly, in the study of Meng and He (2019), 18 out of 42 subjects could not reach 70% performance across three training sessions. In another study, Jeunet et al. (2016a) identified 16.7% of MI-BCI novice users as inefficient because they did not reach the proficiency threshold on performance. While studies have shown that only one hour of BCI training could already induce structural changes in neural plasticity (Nierhaus et al., 2019), and that especially inefficient users could benefit from more training (Meng and He, 2019), the fact remains that some users do not exceed 70% accuracy (Meng and He, 2019). Despite the reported magnitudes in these studies, the factors and neural mechanisms that underlie the inefficiency phenomenon still remain poorly understood, leading to criticism of the concept of “BCI illiteracy,” which suggests that the inability to control the BCI lies with the person (Thompson, 2019). It is crucial to investigate the factors that impact MI-BCI performance among different individuals in order to identify BCI inefficients early on in research because as long as human users cannot modulate their brain activity, even the most advanced classification algorithms will be unable to operate the system sufficiently (Alimardani et al., 2014, 2016).

Several studies have reported psychological, cognitive, and personal factors that influence MI-BCI performance in novice users (e.g., Hammer et al., 2012, 2014; Jeunet et al., 2015, 2016a,b). It has been established that subject-specific differences impact the capability of BCI control (Hammer et al., 2012). Fundamental user characteristics were reported by Randolph (2012), who showed that females are likely to be better performers than males. Additionally, the effect of gender is shown to be modulated by that of the experimenter (Wood and Kober, 2018; Roc et al., 2019; Pillette et al., 2021). Other fundamental characteristics include age, which was reported to influence performance (Randolph et al., 2010) and the dominant hand of the user (Zapała et al., 2020). Moreover, playing a musical instrument and video game experience also increased the likelihood of high BCI performance (Randolph, 2012; Vourvopoulos et al., 2015). Users who were more comfortable with technology tended to perform better in the study of Burde and Blankertz (2006). Experience with mindfulness training also improves BCI performance as it strengthens similar brain activity patterns (Stieger et al., 2021).

In addition, temporal task-related factors as experienced by the subject during the MI task have been shown effective in their performance. For instance, mood was found to influence MI-BCI performance in Nijboer et al. (2008, 2010). Also, motivation of the user during the experiment has been shown to have a significant effect on user performance and behavior during BCI tasks (Nijboer et al., 2008; Bonnet et al., 2013; Alimardani et al., 2014; Sannelli et al., 2019; Škola et al., 2019). Nijboer et al. (2008) found that mastery confidence increased BCI performance, but fear of incompetence was related to decreased performance. While Hammer et al. (2012) could not replicate the same effect, other studies have shown that indirect ways of enhancing motivation such as multi-user BCI games (Bonnet et al., 2013), biased feedback (Alimardani et al., 2014), embodiment of robotic or virtual bodies (Alimardani et al., 2018; Škola et al., 2019; Choi et al., 2020) and immersive technologies (Coogan and He, 2018) can improve users’ performance. Task motivation and engagement could also improve performance by inducing internal competition (Perdikis et al., 2018) or through a continuous pursuit task (Edelman et al., 2019). In a similar way, tiredness and uneasiness were significant performance predictors in the study of Sannelli et al. (2019). These reports are in line with the results of Emami and Chau (2020), showing that BCI performance decreased when mental workload was rising. Another remarkable predictor of BCI performance is the user’s projection of self-performance. In a study where subjects did not receive any feedback, Ahn et al. (2018) showed that users self-prediction of their performance during the task correlated significantly with the observed accuracy after completing more than one run.

The strength of a user’s motor imagery is the core of MI-BCI and is categorized either as kinesthetic or visual imagery of movement. For kinesthetic motor imagery, the subject has to imagine the sensation of executing the movement, whereas for visual imagery, the subject has to visualize the movement execution (Malouin et al., 2007). The success of the MI-BCI system depends on how strongly or vividly the user is able to imagine the movement task to generate the distinctive ERD patterns. There are contradictory reports regarding the impact of kinesthetic versus visual motor imagery on MI-BCI performance; Vuckovic and Osuagwu (2013) found a correlation between MI-BCI performance and both types of imagination as measured by subjective questionnaires, while Marchesotti et al. (2016) showed that only a high score on the kinesthetic scale of the Revised Motor Imagery Questionnaire (MIQ-RS; Gregg et al., 2010) was indicative of high performing MI-BCI users, and the visual scale did not distinguish between high or low aptitude users. On the other hand, Rimbert et al. (2019) found no correlation between MIQ-RS and MI-BCI performance. Hammer et al. (2012) tested the Vividness of Movement Imagery Questionnaire (VMIQ; Isaac et al., 1986) but found no effect on MI-BCI performance.

Related to imagery, several studies reported the effect of spatial ability, which is measured by the Mental Rotation Test and is defined by the ability to rotate an object mentally (MRT; Vandenberg and Kuse, 1978). The relationship between spatial ability and MRT was shown most prevalently by Jeunet et al. (2015) who assessed performance on a three-class Mental Imagery BCI system. A follow-up study by Jeunet et al. (2016a) replicated the effect of spatial ability and showed a correlation between the MRT score and two-class MI-BCI performance of users. The correlation was only significant for BCI peak performance, which is performance at the time when classification accuracy over all trials is at its maximum. However, the study did not find significant results for BCI mean performance, which is calculated as an average of classification accuracy over the total feedback period of all trials (Jeunet et al., 2016a). Another study found that performance on a two-class MI-BCI, which classified rest versus flexion of the arm, correlated significantly with MRT (Pacheco et al., 2017). Furthermore, a pilot-study by Teillet et al. (2016) confirmed that training of spatial abilities could induce an improvement in accuracy on a three-class Mental Imagery BCI.

Other cognitive metrics related to BCI performance include spatial visualization ability (Pacheco et al., 2017), which was measured by the Block Design Test (Wechsler, 1955) and correlated with BCI performance. Memory consolidation of verbal and spatial sequences, measured by the Corsi Block-Tapping Test (Corsi, 1972), was also an important factor in linear models predicting Mental Imagery BCI performance (Jeunet et al., 2015) and Visuospatial memory, measured with the Design Organization Test (Killgore et al., 2005), was found to be higher in better MI-BCI performers (Leeuwis and Alimardani, 2020).

Additionally, personality factors were shown to impact BCI performance by Jeunet et al. (2015); the personality factors tension, abstractness, and self-reliance as measured by sixteen personality factors (16PF; Cattell and Cattell, 1995) were correlated with mental imagery BCI performance but only before correcting for multiple comparisons. Hammer et al. (2012, 2014) already studied effect of personality factors on BCI performance using questionnaires assessing the Big Five personality traits, which are the most commonly used factors to assess personality (Goldberg, 1992) but did not find results. Other personality dimensions that do not rely on the Big Five, such as the temperament profile, have shown promising results; endurance and perseverance were correlated with MI-BCI performance (Zapała et al., 2019). Temperament characteristics are mostly related to psychomotor efficiency; for instance, briskness and endurance have been shown to correlate with better eye-hand coordination (Biernacki and Tarnowski, 2008), which was a predictor of MI-BCI control in Hammer et al. (2012), even though, briskness was not found to correlate with MI-BCI performance in the study of Zapała et al. (2019). Still, personality traits are expected to impact motor imagery, since it is known that certain traits affect imagery abilities, for example in dancers (Budnik-Przybylska et al., 2019).

In sum, the evidence for factors impacting MI-BCI performance is scattered and inconclusive. Additionally, most previous reports suffer from limited subject number. In a meta-analysis of MI-BCI studies, Wierzgała et al. (2018) reported that more than 96% of studies used a subject pool smaller than 10 persons. The current study extends the literature by exploring the relationship that exists between users’ cognitive abilities and personality traits and their MI-BCI performance. More importantly, this study differentiates itself from other reports in the literature in that it has recruited a large sample size and collected a large number of psychological and cognitive variables from subjects. This has improved the statistical power and reliability of the reported results.

## Materials and Methods

### Participants

Fifty-seven novice subjects participated in this study (36 females, 21 males, *M*_Age_ = 20.71, *SD*_Age_ = 3.52). Subjects were all right-handed with (corrected to) normal vision. The study was approved by the Research Ethics Committee of Tilburg School of Humanities and Digital Sciences (REDC #20201003). Prior to the experiment, participants read an information letter and singed an informed consent form.

### Instruments

Personality traits and cognitive abilities were evaluated through five questionnaires and two cognitive tests. Questionnaires were administered using Qualtrics software (Qualtrics, Provo, UT, United States) and cognitive tests were conducted via psychometric toolboxes. In the following sections, each evaluated factor is explained in detail.

#### Questionnaires

**The Demographic questionnaire** evaluated background characteristics such as age, gender and education. Several questions relevant to the present study were added, such as experience and number of hours spent on video gaming, sports participation, and music practice. In addition, participants were asked about their prior experience with BCI.

**The Affinity for Technology Interaction Scale** (ATI; Franke et al., 2019) assesses a person’s tendency to engage in technology interaction actively. ATI included 10 questions and was answered on a 6-point Likert scale ranging from “completely disagree” to “completely agree.”

**The Vividness of Visual Imagery Questionnaire** (VVIQ; Marks, 1973) measures vividness of visual imagery. It quantifies the intensity to which people can visualize settings, persons, and objects in mind. The VVIQ consists of 16 items clustered in four groups in which the participant rates the vividness of the image formed in the mind when thinking about specific scenes and situations. The mental image was rated along a 5-point Likert scale where 5 indicated “perfectly clear and as vivid as normal vision,” and 1 indicated “no image at all.” The original VVIQ was adapted to fit the present study better. Item scoring was reversed to increase interpretability; a higher VVIQ score indicated a higher vividness of the mental image. Moreover, in the original questionnaire each item was imagined once with open and once with closed eyes. Nevertheless, here items were addressed only once with eyes open because visual imagery with closed eyes is meaningless for BCI operation; the participant has to see the motor imagery cue and the feedback to complete the task.

**The Five Factor Personality Inventory** (FFPI; Hendriks et al., 1999) assesses a person’s personality on five dimensions of Extraversion, Autonomy, Orderliness, Emotional Stability, and Mildness. The FFPI consists of 100 questions. Each question was answered on a 5-point Likert scale, ranging from “Strongly disagree” to “Strongly agree.” The key advantage of the FFPI compared to Big Five Personality Inventory (Goldberg, 1992) used by Hammer et al. (2012), is the introduction of anchored scores, where the midpoint is not presented as a true zero, but as the average score in the population (Hofstee and Hendriks, 1998).

**The Questionnaire for Current Motivation** (QCM; Rheinberg et al., 2001) is designed to assess motivational factors in learning and achievement conditions. Nijboer et al. (2010) adapted the questionnaire to assess motivational factors in learning and achievement conditions specified to brain computer interface training. The version by Nijboer et al. (2010) was used in the present study. The motivational factors include Mastery Confidence, Incompetence Fear, Interest and Challenge (Nijboer et al., 2010). The questionnaire consists of 18 statements, each statement contributing to one of the four motivational factors. Items seven, nine, fourteen, seventeen and eighteen were removed from the questionnaire, since these items require familiarity with BCI interaction. This left the questionnaire with 13 statements. The ratings were made on a 7-point Likert scale ranging from “strongly disagree” to “strongly agree.” Item three was a negatively worded statement, hence the rating was reversed. The score for each motivational factor was computed by calculating the mean score of corresponding items. Higher ratings indicate higher motivation levels. To determine if it is justifiable to interpret the QCM test scores after the removal of several items, Cronbach’s alpha (CA; Cronbach, 1951) was calculated and some items were removed from the analysis to increase the CA. The factor Interest displayed a CA of 0.49, factor Incompetence Fear displayed a CA of 0.79, factor Mastery Confidence showed a CA of 0.65 after removing one item (CA before removal was 0.45) and factor Challenge revealed a CA of 0.75 after removing two items (CA before removal was 0.62). A generally agreed rule is that a CA larger than 0.70 indicates an acceptable level of reliability (Tavakol and Dennick, 2011). Therefore, factor Challenge was kept and factors Mastery Confidence and Interest therefore were excluded from the analysis.

#### Cognitive Tests

**FIGURE 1:**
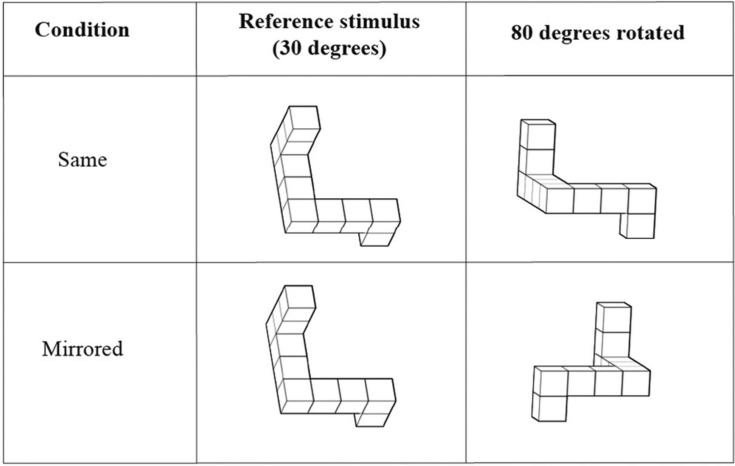
Stimuli in the Mental Rotation Test. A reference stimulus is presented in every trial next to a rotated object. The task of the participant was to determine whether the presented objects were the same or mirrored version of each other. Extracted and modulated from Peters and Battista (2008).

**The Mental Rotation Test** (MRT; Shepard and Metzler, 1971) quantifies the ability to mentally rotate or mirror 2D or 3D representations of objects. An adjusted implementation of Quent (2017) was used to administer the MRT in this study. The stimuli were obtained from Peters and Battista (2008) and were presented in MATLAB 2019a (The MathWorks, Inc.) using the Psychtoolbox (Psychophysic Toolbox Version 3.0.16, Kleiner et al., 2007). Eight reference stimuli were included in the test, each with 5 regular rotations and 5 mirrored rotations. The objects to compare with were rotated 80, 130, 190, 240, or 290 degrees around the x-axis relative to the original object (see Figure 1). The presented reference stimulus was already rotated 30 degrees around the x-axis relative to the original object in the stimulus library of Peters and Battista (2008). This is because the original object showed overlapping legs when displayed on a 2D screen, which made the object unrecognizable.

The test was divided in two blocks of 40 trials, each block containing 20 normal and 20 mirrored stimuli. Subjects were instructed to press the right arrow key if two presented stimuli were the same, and left arrow key if the presented objects were mirrored versions of each other. There were ten practice trials before the start of the test in which subjects received feedback for their performance. However, the actual test trials did not report feedback. Each trial started with a fixation cross that was shown in the middle of the screen for 0.25 s. Subjects had 6 s to respond and if they did not, the trial was registered as incorrect and the test automatically continued to the next trial. The inter-trial interval was 0.25 s. The subject’s MRT score was obtained as the percentage of the correctly answered trials from the total number of the trials in the test.

**FIGURE 2:**
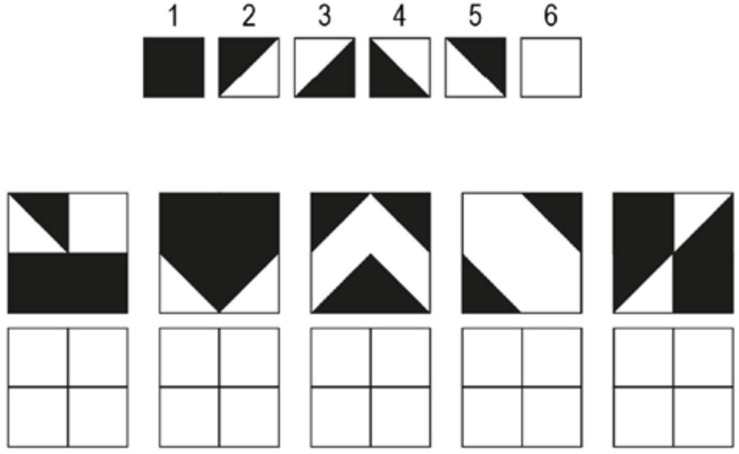
An example of the Design Organization Test (DOT). The task is to reproduce the pattern shown in the square grid using the numerical code given at the top. The participant was instructed to fill out the empty squares at the bottom with the number associated with each piece of the pattern. Extracted and modulated from Killgore et al. (2005).

**The Design Organization Test** (DOT; Killgore et al., 2005) is designed to evaluate visuospatial working memory. The task requires the participant to reproduce black-and-white square grids with a given pattern in them using a numerical code key that translates small pieces of the pattern (see Figure 2). Participants were instructed to reproduce as many patterns as possible within the time limit of 60 s. The DOT consisted of one practice round and two test rounds. Performance score was evaluated by counting the total number of correct responses that the participant produced in both test rounds.

### BCI System and Motor Imagery Task

Electroencephalography signals were recorded from 16 electrodes according to the 10-20 international system (F3, Fz, F4, FC1, FC5, FC2, FC6, C3, Cz, C4, CP1, CP5, CP2, CP6, T7, and T8). A reference electrode was set on the right earlobe and a ground electrode on AFz. EEG signals were amplified by a g.Nautilus amplifier (g.tec Medical Engineering, Austria). A 48-52 Hz Notch filter and 0.5-30 Hz bandpass filter were applied to reduce the noise in the data. The sampling rate was 250 samples/s.

**FIGURE 3:**
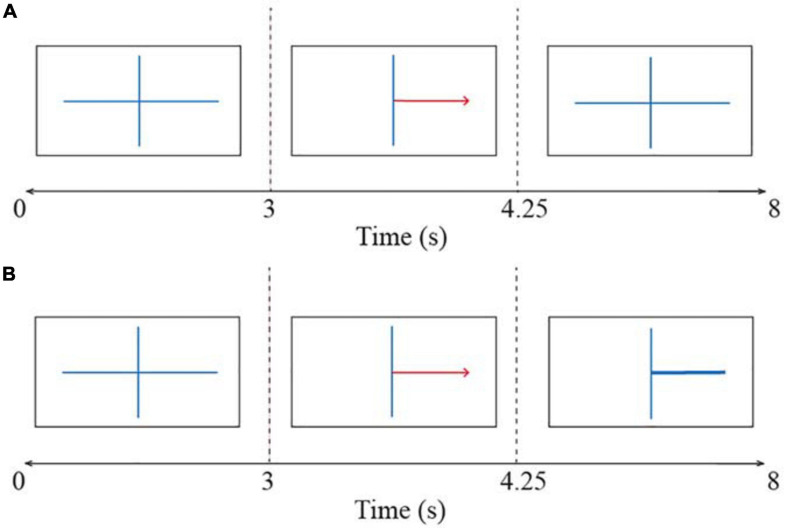
The time course of trials in the BCI task **(A)** during the calibration run and **(B)** during the feedback runs. All trials started with a fixation cross and thereafter a red arrow indicated the direction of the motor imagery task (i.e., left or right). The participant had to imagine a movement for the corresponding hand. In the calibration run participants held motor imagery until the end of the trial. In the feedback runs, a feedback bar indicated the direction of the classifiers’ prediction. A blank screen indicated the end of the trial.

The BCI session consisted of four runs: it started with one non-feedback calibration run followed by three feedback runs in which participants received feedback based on the systems certainty of the classification of their brain signals. Each run consisted of twenty left- and twenty right-hand trials, resulting in 40 trials per run. Each trial took 8 s as can be seen in Figure 3. All trials started with a fixation cross shown for 3 s. Thereafter a red arrow appeared indicating the direction in which the participant had to imagine movement. This arrow was presented for 1.25 s. In the calibration run, the fixation cross was shown again for the rest of the trial duration, which was 3.75 s (Figure 3A). The participant was instructed to hold motor imagery of the corresponding movement during this second fixation. A blank screen indicated the end of the trial. The interval between trials was randomized between 0.5 and 2.5 s. The EEG signals collected in the calibration run were used to extract parameters for a subject-specific classifier in the first feedback run. The trials in the three feedback runs followed the same structure as the calibration run except that after the presentation of the arrow, a feedback bar was shown indicating the direction and certainty of the classifier’s prediction (Figure 3B). The classifier was recalibrated after every run while the subjects took a break. This means that the classifier parameters were recomputed based on the latest run to reflect the subjects’ motor imagery learning throughout the session.

The online classification was conducted by g.BSanalyze, which is a Simulink-based high-speed online processing package (g.tec Medical Engineering, Austria). The classifier relied on Common Spatial Patterns (CSP) algorithm to extract spatial features of event-related (de-)synchronization patterns during motor imagery and to provide a weight of importance to each electrode. These weight vectors were normalized and then served as input to Linear Discriminant Analysis (LDA), which discriminated between left- and right movement imagination during the feedback runs. CSP and LDA are very popular approaches in feature extraction and binary classification of the MI task. LDA has been used in over one third of recent MI-BCI research and CSP is used in 45% of studies (Wierzgała et al., 2018). Trials including artifacts were removed automatically.

### Experimental Procedure

**FIGURE 4:**
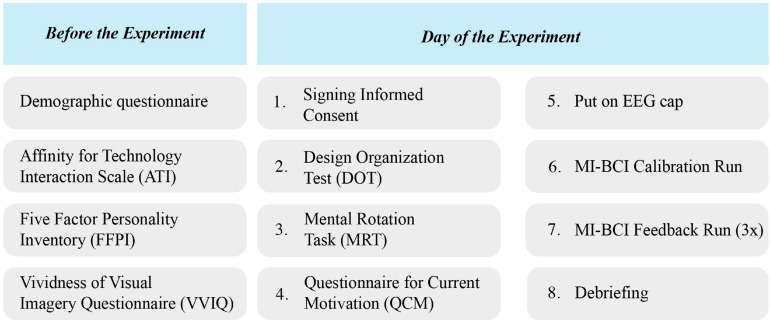
The experimental procedure. Participants completed demographic, ATI, FFPI, and VVIQ questionnaires online before the experiment day. On the day of the experiment, they first completed DOT and MRT tests followed by QCM questionnaire, and then continued to the MI-BCI runs.

The procedure of the experiment is visualized in Figure 4. After application for participation, subjects were informed about the content and procedure of the study by e-mail and were asked to complete four of the questionnaires online: the demographics, VVIQ, ATI, and FFPI.

On the day of the experiment, the participant was seated in front of a desktop in a quiet room. Information on the procedure of the experiment was given to the participant before they signed the informed consent form. First, the DOT was taken using paper and pen and thereafter the MRT and QCM were administered on the desktop before the participant.

Next, the experimenter placed the EEG cap and applied conductive gel. The impedance of all electrodes was kept below 50 kOhm. The experimenter explained that the participant had to avoid unnecessary movements during the experiment and showed live brain activity on the screen to indicate how signals would be contaminated by such events. The experimenter explained the BCI task by elaborating that the subject had to imagine squeezing the left or right hand without tension in the muscles. Squeezing a stressball in the size of a tangerine was given as a reference movement for the subject to focus on. Before starting the calibration run, the subject tried a few trials to ensure they understood the task. During feedback trials, subjects were requested to stay focused on imagining the movement and not get distracted by the feedback because this would decrease their performance. One non-feedback calibration and three feedback runs were completed. The classifier was calibrated between every run based on the data from the previous run. Once all runs were completed, the EEG cap was removed and the subject was debriefed and thanked for participating.

### Data Analysis

The relationship between the variables and BCI performance was evaluated via two analyses; a correlation analysis and a linear regression analysis.

Brain-computer interface error rates were obtained by g.BSanalyze software (g.tec Medical Engineering, Austria). The error rates are defined as the complement of accuracy: Accuracy + Error rate = 1. First, the average BCI error rate for all time points during the 8-s time window of all trials was computed for each of the three feedback runs that the subjects completed. Thus, the calibration run, where the subjects did not receive feedback, was excluded from analysis. Then, in order to specifically obtain the error rates during the MI task period, a segment from second 4.5 to 8 was selected from the trials (see Figure 3B) and the mean of BCI errors was computed over this period. The segment duration was determined by making a consensus between Marchesotti et al. (2016) and Lee et al. (2019). Given that the BCI classifier was recalibrated in each run, the mean of BCI error rates was computed for each run individually and for all runs aggregated. This resulted in four dependent variables per subject to be included in the analysis: one BCI error rate for each run and one BCI error rate for all aggregated runs. The obtained BCI error rates and MRT scores were then checked for gender differences because it is known that these factors can be influenced by gender differences (Peters and Battista, 2008). The difference in motor imagery performance for left and right trials was also investigated, as Zapała et al. (2020) reported variations in performance not only for handedness but also for imagery task laterality.

Correlation analyses were performed between the independent variables and MI-BCI error rates. For normally distributed data, a Pearson correlation (Pearson, 1895) was conducted and for non-normally distributed variables, Kendall coefficients (Kendall, 1938) were obtained. Confidence intervals (CI) for the correlation coefficients were revealed with bootstrapping, which is less sensitive to non-Gaussian distributions. When the bias-corrected CI did not include zero, the correlation was considered significant (Graimann et al., 2002). The number of reproductions was set at 1000.

Stepwise linear regression created models to predict BCI performance in every run. All possible models with one to four predicting and/or moderating variables were estimated for every BCI performance variable. For example, a model with two variables was once calculated with variables as two separate factors and once with the two factors interacting. Normality of residuals was tested with a Shapiro-Wilk test (Shapiro and Wilk, 1965). Linearity assumptions were checked visually. The assumption of homoscedasticity (constant variance) was tested with the Breusch-Pagan test (Breusch and Pagan, 1979; Zaman, 2000). Multicollinearity was tested with Variance Inflation Factor (VIF; Fox and Monette, 1992). VIF was considered critical when higher than five (Akinwande et al., 2015). For each run, the best model that met all these assumptions was selected. The models were checked for outliers using Cook’s distance (Cook, 1977), which has a high sensitivity to outliers (Kannan and Manoj, 2015).

Data analysis was done in R Core Team (2019). All checks for normality of the distributions were done with Shapiro-Wilk test (Shapiro and Wilk, 1965). For all analyses, the significance level was maintained at 0.05.

## Results

**FIGURE 5:**
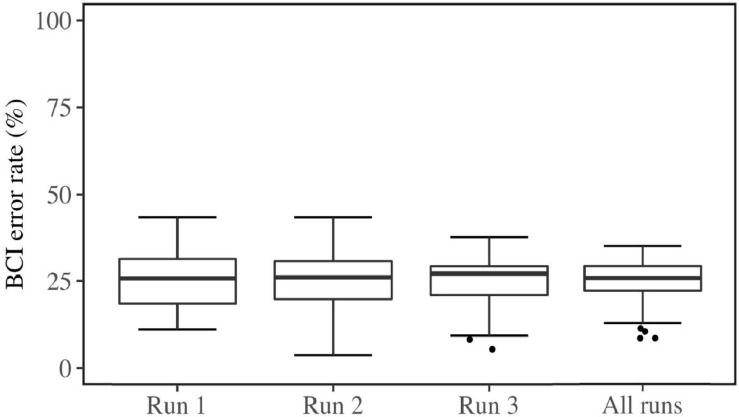
Box plots of mean BCI error rates in every feedback run and all aggregated runs.

After conducting the experiment, two subjects were removed from the data, as they did not meet the criteria for the experiment. Thus, the remaining data included 55 subjects (*M*_Age_ = 20.71, *SD*_Age_ = 3.52, 36 females, 19 males). The demographic questionnaire had 3 missing responses. These were replaced with the median of the category. The ATI questionnaire had one missing value, which was replaced by the middle value of the scale. The FFPI had twelve missing values, seven of which belonged to the same subject. These were replaced by the subject’s mean score across the completed items within the factor pole, as long as the total number of missing items per factor pole for a person was less than 50% (Hendriks et al., 1999), which was not the case for any subject in this study. QCM had no missing values. The mean and standard deviation for each variable (median and interquartile range for non-normal distributions) are summarized in Table 1. BCI error rates in each run and all runs are plotted in Figure 5.

**TABLE 1:** Summary statistics of included variables.

	*Mean*	*SD*	*W*	*p*
BCI Error Rate Run 1	25.471	7.577	0.974	0.273
BCI Error Rate Run 2	24.517	7.922	0.972	0.218
BCI Error Rate Run 3	*Mdn* = 27.237	*IQR* = 8.389	0.957	0.045
BCI Error Rate All Runs	*Mdn* = 25.833	*IQR* = 7.159	0.940	0.008
MRT Correct	0.674	0.131	0.978	0.340
DOT	59.527	7.724	0.981	0.528
ATI	3.955	0.601	0.984	0.671
VVIQ	*Mdn* = 57	*IQR* = 14	0.943	0.011
Interest (not included in analysis)	4.727	0.896	0.975	0.313
Mastery Confidence (CA corrected; not included in analysis)	4.700	0.859	0.959	0.061
Incompetence Fear	3.261	1.158	0.960	0.065
Challenge (CA corrected)	*Mdn* = 6	*IQR* = 0.75	0.913	0.001
Age	*Mdn* = 19	*IQR* = 3	0.677	0.000
Sports	*Mdn* = 3	*IQR* = 2	0.923	0.002
Music	*Mdn* = 0	*IQR* = 1	0.534	0.000
Games	*Mdn* = 2	*IQR* = 5.5	0.651	0.000
Extraversion	*Mdn* = 0.374	*IQR* = 1.637	0.953	0.031
Mildness	2.218	1.044	0.976	0.332
Orderliness	0.494	1.055	0.988	0.860
Emot. stability	0.232	1.309	0.980	0.501
Autonomy	0.952	1.057	0.973	0.241

Brain-computer interface inefficiency within the current sample was evaluated; 10 users did not reach an average accuracy exceeding 70%. Thus the inefficiency rate within this study was 18.18%, which is commonly seen (Allison and Neuper, 2010). The average BCI Error Rate within the efficient users was 23.1% (*SD* = 5.88) and for the group of inefficient users it was 33.4% (*SD* = 1.50). Two users exceeded 90% accuracy. The result of gender comparison showed no significant difference in this sample [*t*(47.27) = −1.42, *p* = 0.16] for BCI Error Rates (*M_F__emales_* = 23.87, *M_M__ales_* = 26.78] and Mental Rotation Scores [*t*(37.22) = 0.21, *p* = 0.83, *M_F__emales_* = 67.64, *M_M__ales_* = 66.84]. The difference in BCI Error Rates between left (*M* = 25.97) and right trials (*M* = 24.49) was not significantly different over all runs [*t*(357.96) = 1.43, *p* = 0.15].

### Outlier Removal

Outliers were tested for both the independent as well as the dependent variables (Leys et al., 2019). Multivariate outliers were identified with Mahalanobis’ distance using chi-square distribution. Mahalanobis’ distance is the distance of a data point from the centroid of the other datapoints, which is calculated as the intersection of all variable means. The distances are interpreted using a significance level of 0.001. One case was found to be a multivariate outlier (*p* = 0.0007) within the dependent variables. The case was particularly high on the BCI Error Rate for the first run obtaining the highest error rate. Therefore, the subject was removed from the linear models for Run 1.

### Correlation Analyses

To account for MI learning effect across runs, four types of BCI Error Rate were calculated; one per each feedback run, and one aggregate measure of all three feedback runs. Thus, the fifteen independent variables had to be correlated with four dependent variables of BCI Error Rate. Bonferroni correction for multiple testing revealed that the significance level should be at 0.003.

**FIGURE 6:**
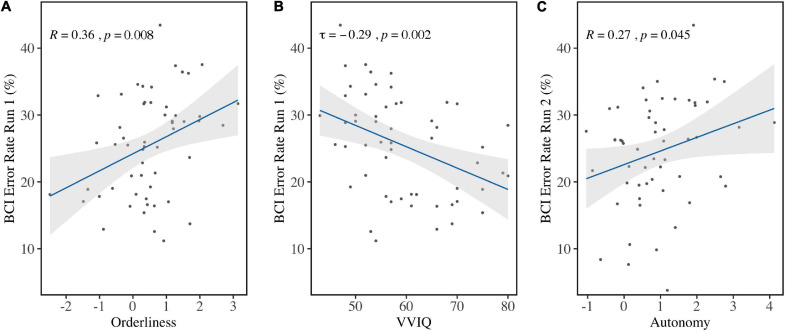
Correlations between BCI Error Rates and individual factors. **(A)** BCI Error Rates in Run 1 were in significant positive correlation with Orderliness and **(B)** in significant negative correlation with VVIQ scores. **(C)** BCI Error Rates in Run 2 were significantly positively correlated with Autonomy before correction for multiple comparison.

Personality factor Orderliness (*M* = 0.49, *SD* = 1.06) correlated with the average BCI Error Rate in Run 1 (*r* = 0.36, *p* = 0.008) before correcting for multiple comparisons (Figure 6A). Bootstrapping revealed the 95% bias-corrected Confidence Interval to be [0.121, 0.520], verifying the result. Run 2 (*r* = 0.14, *p* = 0.29), Run 3 (τ = 0.02, *p* = 0.81), and the aggregate of all runs (τ=0.17, *p* = 0.07) did not show the same effect. This indicated that high ordered personalities performed worse in the first run compared to low ordered personalities. Because it can be expected that performance improves over time, a one-sided t-test was done comparing performance in Run 1 to performance in Run 3 for both high and low ordered personalities. The sample was divided at the median and the accuracies were compared for both groups. This revealed that high but not low ordered personalities improved significantly over runs [*t*(51.40) = 1.82, *p* = 0.04].

Vividness of Visual Imagery (VVIQ; *Mdn* = 57, *IQR* = 14) correlated with BCI Error Rate in Run 1 (τ = −0.29, *p* = 0.002) (Figure 6B) but not in Run 2 (τ = 0.02, *p* = 0.82), Run 3 (τ = −0.09, *p* = 0.33), or the aggregated runs (τ = −0.16, *p* = 0.09). Bootstrapping for the first run revealed the 95% Confidence Interval (bias-corrected) to be [−0.417, −0.140], which verified the result.

Personality factor Autonomy (*M* = 0.95, *SD* = 1.06) was found to correlate with BCI Error Rate in Run 2 (*r* = 0.27, *p* = 0.04) before correcting for multiple comparisons (Figure 6C), but not in Run 1 (τ = 0.08, *p* = 0.37), Run 3 (τ = 0.02, *p* = 0.86), or the aggregate of all runs (τ = 0.08, *p* = 0.37). Bootstrapping on the second run revealed the bias-corrected 95% Confidence Interval to be [0.030, 0.480], confirming the finding. All other variables did not show significant correlation with BCI Error Rates.

### Linear Models

To investigate the predictors of MI-BCI performance even further, linear models were fit including maximum of four out of fifteen numerical and one binary predictors. Stepwise linear regression evaluated the possible combinations of variables and interaction terms for models with one to four predictors. None of the models exceeded 40% explained variance. For every run, the best model was selected based on the adjusted variance ($R_{adj}^{2}$).

Performance in Run 1 was evaluated on 54 participants after rejecting one outlier. The BCI Error Rate was best predicted by Gender + Emotional Stability + Orderliness + VVIQ ($R_{adj}^{2}$ = 0.347, *p* < 0.001). The model revealed that being male increased BCI Error Rate [*b* = 5.643, *t*(49) = 3.080, *p* = 0.003], and a higher Emotional Stability score decreased the error rate [*b* = −1.488, *t*(49) = −2.304, *p* = 0.026]. A lower score on Orderliness [*b* = 2.476, *t*(49) = 2.914, *p* = 0.005] decreased BCI Error Rate as did a higher Vividness of Visual Imagery [*b* = −0.338, *t*(49) = −3.829, *p* < 0.001].

In Run 2, the BCI Error Rate was best predicted by Autonomy ($R_{adj}^{2}$ = 0.056, *p* = 0.006). A more autonomous personality led to increased BCI Error Rate [*b* = 0.036, *t*(53) = 2.057, *p* = 0.045].

For Run 3, it was not possible to construct a significant predicting model. The analysis of variance comparing the explained variance (sum of squares of regression) to the unexplained variance (sum of squares of errors) was not significant.

Finally, the model best predicting BCI error rate in all aggregated runs was composed of Gender + Autonomy + Emotional Stability + VVIQ ($R_{adj}^{2}$ = 0.018, *p* = 0.011). Being a male increased BCI Error Rate [*b* = 3.857, *t*(50) = 2.085, *p* = 0.042] and a higher autonomous personality predicted a higher BCI Error Rate [*b* = 1.936, *t*(50) = 2.203, *p* = 0.032]. Emotional Stability was not a significant predictor [*b* = −1.304, *t*(50) = −1.996, *p* = 0.051]. Vividness of Visual Imagery negatively predicted BCI Error Rate [*b* = −0.306, *t*(50) = −3.061, *p* = 0.004].

## Discussion

This study aimed to establish the factors that impact MI-BCI performance in novice users. Participants conducted three consecutive MI runs and their personality traits and cognitive abilities were measured. Correlation analyses showed that Vividness of Visual Imagery was negatively correlated with MI-BCI Error Rate in the first run of the experiment, while in the same run personality factor Orderliness correlated positively with MI-BCI Error Rate. The personality factor Autonomy correlated positively with BCI Error Rate in the second run of the experiment. From a predictive standpoint, linear models were found that could predict the BCI Error Rate in the first, second and the aggregate of all runs based on factors such as Gender, Vividness of Visual Imagery, personality traits and Emotional Stability. In the following, we further discuss our results for each cognitive and psychological factor.

### Vividness of Visual Imagery

The obtained scores in the Vividness of Visual Imagery Questionnaire (VVIQ) were shown to be correlated negatively with the mean BCI Error Rate in the first run. This result indicated that a more vivid imagery related to a lower Error Rate, i.e., a better MI-BCI performance. The negative coefficient in the linear model for aggregated runs indicates that, while the correlation was not significant in the second and third runs, visual imagery is an essential factor in MI-BCI performance. It was already established by Vuckovic and Osuagwu (2013), and Marchesotti et al. (2016) that kinesthetic imagery scores relate to BCI performance. The results for visual imagery were questionable in the past since Vuckovic and Osuagwu (2013) reported a positive relationship, but Hammer et al. (2012); Marchesotti et al. (2016) did not find a significant relationship. The result of this study underscores the importance of visual imagery as a proxy for kinesthetic imagination.

### Personality Factors

The study of Jeunet et al. (2015) is the only study that reports significant effects of personality factors on MI-BCI performance. While earlier studies by Hammer et al. (2012, 2014) did not find a relationship between personality variables and BCI performance, a recent study by Zapała et al. (2019) showed that endurance and perseverance, which are temperament traits, were correlated with MI-BCI performance. This provided further support that MI-BCI control is impacted by the user’s personality traits.

The current study assessed personality traits with the Five Factor Personality Inventory (FFPI) (Hofstee and Hendriks, 1998), while Jeunet et al. (2015) used the 16 Personality Factors (16PF; Cattell and Cattell, 1995). To compare our results with the results of Jeunet et al. (2015), the relations between various personality inventories were explored. Jeunet et al. (2015) found that Abstractedness correlated positively with BCI performance. Abstractedness contributes negatively to 16PF global factors Tough-Mindedness and Self-Control (Cattell and Cattell, 1995). Self-Control positively relates to Conscientiousness (Herrmann and Pfister, 2013) in the Revised NEO Personality Inventory (NEO-PI-R; Costa and McCrae, 1992), which was positively correlated with FFPI factor Orderliness (Hendriks et al., 1999). Thus, the relationship between Abstractedness and Orderliness was concluded to be negative, and the hypothesis was deduced that Orderliness should negatively correlate with BCI performance and thereby positively with BCI Error Rate.

Our results supported the above hypothesis: Orderliness was found to correlate positively with BCI Error Rate in the first run, meaning that a higher score on orderliness scale was associated with a lower accuracy on MI-BCI during the first run. One of the characteristics of conscientious personalities is the lack of fantasy, which may be indicative of a decreased ability to visualize mentally (McCrae et al., 1986). Conscientiousness is also negatively related to creativity (Jirásek and Sudzina, 2020). Creativity is linked to the ability of mental imagery (May et al., 2020), and possibly a lack of creativity in conscientious personalities might also consequence their inferior MI-BCI performance. The data was explored further to reveal why this trait only affected performance in the first run. We found a learning effect in subjects with high Orderliness; their performance improved in the runs that followed while low ordered personalities kept constant. This may be explained by the fact that higher ordered individuals are more achievement-oriented learners (Chamorro-Premuzic et al., 2007) and thereby may have improved their performance more easily when feedback was provided.

Abstractedness also contributed negatively to Tough-Mindedness in the study of Cattell and Cattell (1995). Tough-Mindedness is negatively correlated with the NEO-PI-R factor Openness to Experience (Herrmann and Pfister, 2013). Openness correlated negatively with FFPI Orderliness and sometimes correlated positively with Autonomy according to Hendriks et al. (1999). This indicated again that Abstractedness should correlate negatively with Orderliness. Openness and Autonomy correlated positively, and thus Abstractedness and Autonomy are expected to correlate positively. From this, the hypothesis for the current study was deduced that there could exist a positive relationship between Autonomy and BCI performance. This hypothesis was also partially supported by the results of the current study, where it was found that Autonomy correlated positively with BCI Error Rate in the second run. Autonomy was also a significant positive predictor in models predicting BCI Error Rate. These results indicate that a person with a higher score on autonomy is likely to perform worse at motor imagery BCI.

The difference in these results may be due to the unstable correlation between NEO-PI-R Openness and Autonomy, which was reported by Hendriks et al. (1999). Autonomy is known to increase motivation and learning efficiency in general (Lotte et al., 2013), which leads to the expectation of a positive relationship between autonomy and MI-BCI performance. Jeunet et al. (2015) argued that incorporating more autonomy in the task may increase performance for highly autonomous BCI users, and this might reduce the negative effect of autonomy found in this study. In future studies, this should be quantified and further explored.

Jeunet et al. (2015) found that Tension correlated negatively with BCI performance. Tension is a sub-factor of 16PF global factor Anxiety that correlates positively with NEO-PI-R factor Neuroticism (Herrmann and Pfister, 2013), which in turn correlates negatively with FFPI factor Emotional Stability (Hendriks et al., 1999). Thus, it was concluded that Tension is negatively correlated with Emotional Stability, and thereby a positive correlation between Emotional Stability and BCI performance was hypothesized. This is in line with the found negative predictor coefficient for Emotional Stability impacting the BCI Error Rate in the first run and also in predicting error rate on the aggregated runs. Emotionally stable personalities are calm, well-balanced, and can deal with stress, while emotionally unstable personalities are emotional, sensitive, and easily overwhelmed (Hofstee and Hendriks, 1998). An explanation may be that emotionally unstable personalities were overwhelmed by the experiment and therefore performed poorly.

Another factor found by Jeunet et al. (2015) to impact BCI performance is self-reliance, which negatively contributes to 16PF Extraversion (Cattell and Cattell, 1995). As expected, 16PF Extraversion correlated positively with NEO-PI-R Extraversion (Herrmann and Pfister, 2013), which positively correlated with FFPI Extraversion (Hendriks et al., 1999). Thus, it was concluded that self-reliance is negatively correlated with Extraversion, and therefore the hypothesis was that Extraversion would correlate negatively with BCI performance. However, we did not find any result that would support this hypothesis. This inconsistency in the findings of the current study compared to that of Jeunet et al. (2015) can be explained based on the different experimental design the was employed in the two studies. While this study conducted only one BCI session, Jeunet et al. (2015) repeated the same experiment in six different sessions, which may have affected the participants’ arousal levels. Introverted personalities, who prefer quietness and a lower mental workload might have been fine with such repetition of the same task while extroverted personalities, who are energized by bustling environments, got bored by it (Hofstee and Hendriks, 1998).

### Spatial Abilities

Jeunet et al. (2015) found significant results for visuospatial memory, which was measured by the Corsi Block test. The current study incorporated the Design Organization Test (DOT), which also quantifies visuospatial memory. The Corsi Block test was replaced because its underlying constructs are insufficiently understood (Berch et al., 1998; Cornoldi and Vecchi, 2004). Pacheco et al. (2017) used Block Design Test as an alternative and showed its relationship with MI-BCI performance. However, the Block Design Test consumes a lot of time, contrary to the DOT which takes only a few minutes. In this experiment, we opted for DOT as multiple cognitive measures had to be collected on the same day. Nonetheless, we did not find any significant correlation between DOT scores and the BCI performance, although our earlier results showed that high aptitude BCI-users performed better on the Design Organization Test (Leeuwis and Alimardani, 2020). A more reliable test should establish the contribution of visuospatial factors in future research.

Similarly, the strong results of Jeunet et al. (2015, 2016a) regarding the correlation between Mental Rotation Test (MRT) and BCI performance raised the expectation of an effect of MRT scores in the current study. However, we did not find a relationship between BCI performance and MRT scores. Previous studies (Jeunet et al., 2015, 2016a; Pacheco et al., 2017) used the MRT as implemented by Vandenberg and Kuse (1978) which shows multiple objects simultaneously, whereas the current research used the implementation by Shepard and Metzler (1971), which is a pairwise object presentation. This choice was made because the results with implementation of Vandenberg and Kuse (1978) tend to be affected by gender differences (Peters and Battista, 2008). In addition, the BCI classifier in Jeunet et al. (2015) classified mental rotation as one of the mental tasks conducted by the subjects, which may have driven the direction of their results. Therefore, the relationship between spatial abilities and MI-BCI performance should be further validated in future experiments. It would be interesting to look also at reaction times of the test, as Darvishi et al. (2018) showed that scores on the reaction time test are related to BCI performance.

### Fundamental Characteristics

Gender was expected to influence performance: being a woman, in general, coincides with better performance (Randolph, 2012). Cantillo-Negrete et al. (2014) also proposed a gender-specific BCI system. Their study showed that women perform better and that both men and women users benefit from a BCI classifier that is trained with the data of their gender group rather than a subject-independent BCI system. Our results confirmed that indeed being a woman was a predictor of lower BCI Error Rates in the first run and aggregate of all runs. This is equal with the distribution of gender effects in Roc et al. (2019) where males performed worse in the first runs compared to later ones while females tended to perform more steadily: starting with a higher accuracy compared to men but not increasing their performance any further. The distribution of genders was not equal in the current study, which may have given a skewed result, although this was corrected by using Welch’s *t*-test. In addition, differences in gender performances might have been prevailing because all experimenters in the current experiment were females. While Roc et al. (2019) suggested that female experimenters might positively influence subjects’ performance, Pillette et al. (2021), using the same data, reported that the evolution of MI-BCI performance is dependent on the interaction between experimenters’ and subjects’ gender as well as subjects’ tension level and that it cannot be firmly said that female experimenters benefit all subjects. This is while Wood and Kober (2018) found that female participants trained by female experimenters performed significantly worse than those trained by male experimenters. These inconsistent findings hint that there is a complex relationship between BCI performance and gender of both subjects and experimenters, which deserves further investigation in the future research.

Age was expected to impact BCI performance based on the study by Randolph et al. (2010). However, no significant results were found. The distribution of age in the current study was not normal and there was little variance. The sample mainly consisted of young people (*Mdn* = 19) where a few older subjects pushed the variance. Other studies, such as Rimbert et al. (2019), also found no significant effect of age. Therefore, its relation with BCI performance remains unconfirmed.

Sport participation did not show any effect on BCI performance in the current study, however it has been shown that physical ability and mental imagery quality are closely related (Martin et al., 1999), and that regular physical practice can improve motor imagery (Rimbert et al., 2019). This indicates that the relationship between playing sports and BCI performance must further be elaborated in future studies.

Game playing was expected to have a positive influence on BCI performance because Randolph (2012) found an effect of playing games per week. Vourvopoulos et al. (2015) also found that gaming experience can enhance BCI performance and strengthens the underlying brain activity. Additionally, Hammer et al. (2012, 2014) found an effect of two-hand coordination test, which measures speed and coordination accuracy in hand movements with a joystick and is especially important when playing games. The current study did not find any significant results for video gaming in relation to BCI performance. This might be due to the very skewed distribution: there were eight outliers corresponding to participants who played more than 10 h per week (compared to the median of 2 h per week). There was a robust relationship between gender and playing games, which may have induced the skewness of the distribution because genders were not equally distributed across the sample.

Music practice was expected to have a positive influence on BCI performance as music modulates the mu rhythm (Randolph, 2012), which drives the classification of motor imagery. However, Rimbert et al. (2019) found no significant effect, nor did this study. This may be explained by the number of music players in the current study: the median hours per week was zero.

Users that are comfortable using technology were shown to perform better on BCI by Burde and Blankertz (2006), however the current study found no such relationship. The Affinity for Technology Interaction Scale (ATI) did have a correlation with music, games, and gender; thus, this collinearity may explain why it did not appear in any of the models. Nevertheless, no relationship was found between ATI and BCI Error Rates. It should be noted that Burde and Blankertz (2006) used a different questionnaire that focused more on the feeling of control when using technology, contrasting the employed ATI in this study which measures interest in technology. This distinction may explain the different results.

### Temporal Factors

The users’ motivation during the experiment was expected to impact MI-BCI performance based on the study of Nijboer et al. (2008), who found that mastery confidence increased BCI performance and that higher fear of incompetence was associated with decreased performance. However, the current study could not confirm these results, nor did Hammer et al. (2012). The current study removed five questions from the original QCM questionnaire because they were only applicable if the participant had already conducted a BCI task before. The manipulations of the questionnaire might have reduced the validity of this questionnaire, thereby making it a poor measure for novice BCI-users. Future studies investigating the effects of motivation may not only passively measure one’s motivation, but also actively induce it. Examples of enhancing motivation include better embodiment by using robotic or virtual bodies (Alimardani et al., 2018; Škola et al., 2019), multi-user BCI games (Bonnet et al., 2013; Daeglau et al., 2020), or feedback that incorporates a positive bias (Alimardani et al., 2014).

### Limitations

When comparing results of the studies discussed, variation in the study design must be taken into account. We mainly compared our results to the findings of Jeunet et al. (2015) who employed similar variables as this study did. However, there were differences in the two studies including the number of sessions; Jeunet et al. (2015) conducted six sessions on six different days, while the current study only included one session. In addition, Jeunet et al. (2015) employed a three-class mental imagery BCI, which demanded mental rotation and mental arithmetic to be performed. This may have yielded different results compared to the two-class motor imagery BCI employed in this study because mental rotation and mental arithmetic work on different brain activity than does motor imagery task. In previous research, more than 50% of the studies have been employed on the two-class left versus right motor imagery (Wierzgała et al., 2018).

The participants in the current study were employed via university participant pool and convenience sampling, which resulted in a skewed distribution of gender and age in the sample. This may suggest that the sample is not generalizable to other populations such as motor-impaired or elderly users, who are traditionally the target audience of MI-BCI development. However, it has been shown that the physiological changes resulting from BCI training may improve performance in both healthy individuals and patients (Perdikis et al., 2018; Edelman et al., 2019) and in several studies BCI learning did not significantly differ between stroke patients and healthy users (Kober et al., 2015; de Castro-Cros et al., 2020). Additionally, recent studies indicate that the target audience of MI-BCI has extensively expanded to non-medical users, for example gamers (Kerous et al., 2018). Therefore, this research applies to a wide range of potential future users and thereby has broad impact for the adoption of MI-BCI in society.

Furthermore, many of the questionnaires were not administered on the day of the experiment but were sent beforehand. This was done in order to reduce the required time for the experiment and hence reduce exhaustion of the participants during the BCI session. The variety in devices and environments in which participants filled out the questionnaires may have affected their answers; a lack of control by the experimenter, social control by friends or family, being in a rush or other external factors may have influenced the answers given on the FFPI, ATI, and VVIQ questionnaires.

In addition, the explanation given to the subjects about the motor imagery task did not explicitly state whether the subject should imagine kinesthetic or visual movement, which is known to make a difference; kinesthetic imagery produces greater activation of the primary motor cortex and supplementary areas because it is more intuitive compared to the visual imagery of movement (Burggraaf et al., 2016). Furthermore, the current study included only 120 feedback-trials, which might have reduced the validity. Typically, studies have 160 to 320 trials to produce reliable results (Müller-Putz et al., 2008). When performance was evaluated per run, this number was reduced to forty trials.

Another limitation in the current study that is also found in Jeunet et al. (2015) is the number of statistical tests. Correction of multiple comparisons wiped out almost all results of Jeunet et al. (2015). Similarly, in the current study, Bonferroni correction immediately disarmed almost all correlations. Bonferroni reduces false positives but also produces false negatives by its conservativeness. As a check on robustness of the results, the bootstrapping confidence interval for the correlation coefficients was provided. In addition to correlation analyses, this study employed a stepwise linear regression and evaluated over 100,000 linear models. The construction of four different stepwise linear regression models selecting combinations out of fifteen possible predictors, gives an abundance of possibilities. Consequently, the results reported above are likely to contain false positives. One advantage of including this many variables is the reduced chances of exogeneity: few factors could influence BCI performance that were not assessed by the current study.

### Future Research

It is unclear whether BCI inefficiency reflects a failure on behalf of the subject or BCI system and whether this distinction is meaningful (Allison and Neuper, 2010). Therefore, the authors of the current study empathize that all results should be interpreted under the methodological guidelines used in this particular experiment. The recalibration of the left- versus right-hand motor imagery classifier between every run is an important aspect of this.

So far, studies have been learning on little data: most studies tested under twenty participants that perform a maximum of six sessions. To make more robust predictions, it is needed to perform a large-scale study in which subjects are trained for a more extended period. This would enable researchers to discriminate individual traits from states and observe when performance improves; how many runs it takes and what factors are important. In order to gain better results, the authors advise further research with an updated BCI paradigm to make it a reliable extension of the current state of the art. There are multiple improvements in the field that suggest motor imagery learning among novice BCI users; for example, it has been shown that realistic feedback from humanlike bodies and the feeling of embodiment improves the modulation of brain activity needed for motor imagery (Alimardani et al., 2018; Penaloza et al., 2018; Choi et al., 2020). Furthermore, visual guidance in virtual reality (Liang et al., 2016; Coogan and He, 2018), gamification (de Castro-Cros et al., 2020), and multimodal visual-haptic feedback (Wang et al., 2019) can improve learning of MI-BCI. Improving the training conditions might reveal a more robust difference between (in-)efficient learners and thereby provide more valid evidence for the impacting variables on MI-BCI.

In the same vein, Thompson (2019) proposed user-centered approaches instead of the one-fits-all approach. Here, users themselves define usability and thereby researchers are enabled to focus more on the issues that are experienced by the users (Thompson, 2019). For example, Cantillo-Negrete et al. (2014) proposed a MI-BCI system specifically designed for male or female users. It is essential to focus research on the user, since advanced technology alone will not be sufficient for a user to operate the system when (s)he is unable to generate the MI-specific brain patterns (Alimardani et al., 2014, 2016).

The current study focused only on online classification accuracy. There are several arguments that favor this approach as it is evaluated in similar research (Jeunet et al., 2015). Wierzgała et al. (2018) reported that the minority of studies were conducted online, i.e., during real-time control (only 4.9%), while in our opinion, real-time performance, which is the main source of feedback can greatly influence the user performance in the following trials. In this process, personality factors or skill level of the user can moderate the subjects’ interpretation of the feedback; some might benefit from positive feedback, while others may not (Barbero and Grosse-Wentrup, 2010; Alimardani et al., 2014; Penaloza et al., 2018). By evaluating the online performance, this confounding effect is accounted for. However, offline analyses of this dataset would be encouraged to further evaluate the psychological and cognitive factors that might impact offline performance. This would include extracting mu-suppression from the C3 and C4 electrodes (Penaloza et al., 2018) and evaluating the sensorimotor rhythms that underlie the classifiers’ accuracy scores.

Future research should look into other EEG measures that account for BCI aptitude and performance. For instance, recent studies have proposed that connectivity measures can uncover the underlying brain activity in (in-)efficient users (Lee et al., 2020) and that coherence measures can improve accuracy for inefficient users as compared to CSP-dependent classifiers (Zhang et al., 2019). In addition, Nierhaus et al. (2019) showed that functional connectivity in the brain networks increased already after 1 h of BCI training. Thus, future research might focus on extending the role of functional connectivity in generating the brain activity needed for MI-BCI control. This could provide a new predictor of BCI performance and increase insights in the networks needed for successful modulation of the sensorimotor rhythms.

Furthermore, the BCI classifier in this study relied on a classic machine learning approach, however, some studies have shown that BCI classification may benefit from more advanced approaches such as deep learning as compared to the traditional CSP+LDA method (Ko et al., 2020). Especially, low aptitude users seem to benefit from deep learning classifiers more (Stieger et al., 2020), thereby making it a promising tool for solving MI-BCI inefficiency. Further investigation of this topic might improve MI-BCI control for inefficient users.

Finally, another field of research lies in merging and analyzing the growing body of data on the topic. For example, Ko et al. (2020) merged dataset of Cho et al. (2017) and Lee et al. (2019), datasets that are also used by Lee et al. (2020) and Velasquez-Martinez et al. (2020). Merging public datasets with similar BCI paradigms could be a cost-effective way of examining the relationships between and within studies even further. In the same vein, future research can employ a new group of novice BCI users for further testing of the model proposed in this research, thereby validating the findings and extending our work to find more robust user characteristics of BCI inefficiency.

### Contribution to the Field

To summarize, the question remains if motor imagery BCI performance can be predicted. This study contributed to the literature by confirming effects of visual imagery and orderliness on MI-BCI performance. In addition, a new result was found for personality trait autonomy and its negative correlation with BCI performance. It was established that females tend to perform better and that better emotional stability is a predictor of improved BCI performance. The number of participants and the controlled experiment environment make this a reliable study. The unique combination of measured variables, and the use of the regular right-versus left-hand imagination instead of mental imagery tasks distinguishes this study from previous research.

## Conclusion

This study attempted to answer the question whether MI-BCI in novice users can be predicted by their psychological and cognitive measures. Significant correlations were found between BCI performance and personality factors Orderliness and Autonomy as well as Vividness of Visual Imagery. Additionally, multiple linear models were fit to the data in which a combination of Gender, Emotional Stability, Vividness of Visual Imagery and personality factors Orderliness and Autonomy were found as significant predictors of BCI performance. The relationships found in this study can be contributing factors in future studies that will assess BCI trainings for multiple sessions and an increased number of participants.

## Data Availability Statement

The raw data supporting the conclusions of this article will be made available by the authors, without undue reservation.

## Ethics Statement

The studies involving human participants were reviewed and approved by the Research Ethics Committee of Tilburg School of Humanities and Digital Sciences (REDC #20201003). The patients/participants provided their written informed consent to participate in this study.

## Author Contributions

NL and AP conducted the experiment and performed the data analysis under supervision of MA. NL wrote the manuscript with input from MA. All authors designed the research.

## Conflict of Interest

The authors declare that the research was conducted in the absence of any commercial or financial relationships that could be construed as a potential conflict of interest.
